# The Development of a New IFOG-Based 3C Rotational Seismometer

**DOI:** 10.3390/s21113899

**Published:** 2021-06-04

**Authors:** Yuwen Cao, Yanjun Chen, Tong Zhou, Chunxia Yang, Lanxin Zhu, Dingfan Zhang, Yujia Cao, Weiyi Zeng, Dong He, Zhengbin Li

**Affiliations:** 1 State Key Laboratory of Advanced Optical Communication Systems and Networks, School of Electronics Engineering and Computer Science, Peking University, Beijing 100871, China; chenyanjun@pku.edu.cn (Y.C.); yangchunxia@pku.edu.cn (C.Y.); zhulanxin2020@stu.pku.edu.cn (L.Z.); zhangdingfan@pku.edu.cn (D.Z.); zengweiyi@pku.edu.cn (W.Z.); hedong@pku.edu.cn (D.H.); lizhengbin@pku.edu.cn (Z.L.); 2 School of Software and Microelectronics, Peking University, Beijing 102600, China; zhoutong96@pku.edu.cn; 3 Institute of Geophysics, China Earthquake Administration, Beijing 100081, China; caoyj@cea-igp.ac.cn

**Keywords:** rotational seismometer, three-component, sagnac effect, fiber optic gyroscope

## Abstract

For many years, seismological research mainly focuses on translational ground motions due to the lack of appropriate sensors. However, because of the development of devices based on Sagnac effect, measuring rotational waves directly comes available. In this work, a portable three-component broadband rotational seismometer named RotSensor3C based on open loop interferometric fiber optic gyroscope (IFOG) is designed and demonstrated. Laboratory tests and results are illustrated in detail. The self-noise ranging from 0.005 Hz to 125 Hz is about $1.2\times 10^{-7}\;{\mathrm{rads}}^{-1}/\sqrt{\mathrm{Hz}}$, and with the harmonics compensation the scale factor variation over $\pm 250^{\circ }/s$ is lower than 10 ppm (parts per million). The misalignment matrix method is adopted to revise the output rotation rate. In a special near field experiment using the explosive source, the back-azimuths and phase velocity are estimated by the recorded acceleration and rotation rate. All the results prove the practicability of this new rotational sensor.

## 1. Introduction

The rotational effects in the great earthquakes attract researchers to develop seismological theory and find out the physical nature of seismic waves. In theory, to fully characterize the motion of a deformable body at a given point in the context of infinitesimal deformation, one needs three components of translation, six components of strain, and three components of rotation [1]. However, the effects of rotational motions are still ignored in modern observational seismology, as the results of the difficulties in measuring rotational motions and the widespread belief that they are insignificant [2]. With the deeper learning of rotational ground motions, more and more researches suggest that they are not negligible and may contribute to co-seismic structural damages [3]. Recently, because of the development of devices based on Sagnac effect, measuring rotational seismic waves directly becomes available, and these sensors display a lower flat frequency range for measurement [4]. Therefore, rotational seismology has been rapidly developed, attracting much attention from many communities to explore additional valuable information in rotational ground motions [5], which promotes the applications in seismology and civil engineering structures [6,7]. Rotational ground motions on the far field have been successfully measured at sites [8]. The rotational measurements on the near field indicate that rotational ground motions are 10 to 100 times larger than that expected from the classical elasticity theory [9,10], and these rotational motions induces the torsion response of buildings due to the eccentricity [4]. Therefore, in certain cases rotational seismic waves may cause larger damage to buildings.

The common approaches to observe rotations include mechanical devices, seismic arrays, IFOGs and ring laser gyroscopes (RLGs). According to their measurement characteristics, they can be divided into two groups of sensors. One group of sensors, such as mechanical devices and seismic arrays, are based on classical translation or acceleration recording, which can only detect rotation indirectly. The other group of sensors, including IFOGs and RLGs, are based on Sagnac effect and utilize an inertial mass as the reference. Comparing with the first group of sensors, these two kinds of optical devices are completely insensitive to translational motion and able to measure rotational motions directly [11]. Moreover, they are also more accurate, making them the perfect candidates for the measurement of strong motion. Although the IFOGs are less sensitive to record rotational signals of distant earthquakes compared with large RLGs, as for the strong near field seismic events, the IFOGs are the more available sensors and more portable in size, since the RLGs are too sensitive and would go out of alignment immediately after the first shock [12]. In addition, the frequency response of IFOGs is only limited by the travel time of light through the optical fiber loop, which means that IFOGs inherently have a flat broadband frequency response in theory and are easier to achieve a relatively flat frequency range in practice [13]. Moreover, IFOGs are cheaper than the ring lasers to satisfy the sensitivity requirement of near-field rotational sensors. Based on the above characteristics, IFOGs are considered to be the preferred solution to realize the portable, reliable, and high sensitivity broadband rotational seismometer [14].

This paper is organized as follows: In Section 2, a portable three-component (3C) broadband rotational seismometer named RotSensor3C IFOG-based is designed and demonstrated. A tentative application of the RotSensor3C is presented and discussed in Section 3. Section 4 presents the main conclusions and prospects.

## 2. Three-Component Rotational Seismometer

### 2.1. Working Principal

As mentioned before, considering the portability, reliability and sensitivity, IFOG is the preferred solution for seismological applications. The operation of the IFOG is based on the Sagnac effect [15]. In an IFOG, the beam splitter separates the input beam into two counter-propagating light beams inside the closed fiber coil, and a phase shift will be caused by the rotational motions. The Sagnac phase shift $\phi_{s}$ is proportional to the rotation rate Ω and can be expressed as [16]:

(1) $$\phi_{s}=\frac{2\pi LD}{\lambda c}\Omega ,$$

where *L* and *D* are the length and diameter of the closed fiber coil, respectively; λ is the central wavelength, and *c* is the speed of light in vacuum.

**Figure 1:**
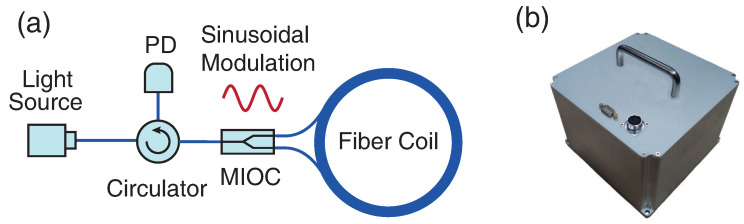
(**a**) Schematic diagram of the IFOGs adopted in the RotSensor3C; (**b**) picture of the RotSensor3C.

Due to the requirements of portability and sensitivity, three 4-km-long polarization-maintain (PM) fiber coils (average diameter of 145 mm) are utilized and placed orthogonally in RotSensor3C with open-loop configuration shown in Figure 1a. An amplified spontaneous emission (ASE) light source (average light intensity $I_{0}$ = 15 dBm, central wavelength $\lambda_{c}$ = 1550 nm, spectral width Δλ = 40 nm, rectangular spectral) with degree of polarization (DOP) below 1.0% is adopted. Then the input beam comes to the fiber circulator which is used for a better power budget than the fiber coupler to guide the propagation direction. Besides, a multifunctional integrated optical chip (MIOC) is adopted and the light beams are polarized with a high polarization extinction ratio ($\varepsilon^{2}<80$ dB, typical) and split into two beams (50:50). Then the two split beams will be modulated by sinusoidal signals at third-order eigen frequency of 73.5 kHz to reduce the thermal phase noise [17] and counter-propagate inside the closed fiber coil. The interfered light beams come back to the photodetector (PD) converting the optical signals to electrical ones. The electrical signal will be processed and demodulated in the electrical system and the output rotation rates are digital format saved finally. In order to avoid the data timing issue between three-component rotational sensors and other translational seismometer, our system is connected to the GPS module to receive the 1 PPS (pulse per second) signal which can be used for precise time synchronization, so each output rotation rate has its own precise time (error < 10 s, typical) saved together. All above optical devices and supplementary electrical control system are packed into the shell in Figure 1b of size 190 mm (L) × 190 mm (W) × 165 mm (H) to guarantee the reliability and portability under complex situations.

### 2.2. Laboratory Tests of 3C Rotation Sensors

Before deploying this 3C rotation sensors in practical applications, detail laboratory tests, including stability over time, scale factor linearity and sensor-axis orthogonality, need to be completed to get the preliminary technical specifications.

#### 2.2.1. Stability over Time

The Allan variance is a general method for analyzing the performance of inertial sensors [18]. For practical purposes, the Allan deviation (ADEV) σ(τ) vs. averaging time τ is usually adopted [19], which is also widely used to estimate the IFOGs. For the evaluation of the performance of IFOGs, angle random walk, bias instability and self-noise level are the three most important parameters, which determine the short-term noise, the long-term drift and the minimum detectable amplitude, respectively [20]. However, as a seismometer, the angle random walk and self-noise are more significant for recording signals since the near-field earthquake would not last for a long time.

**Figure 2:**
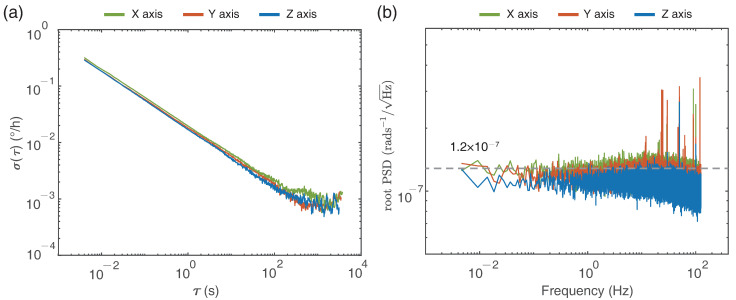
(**a**) Allan deviation curves and (**b**) root power spectral density of the rotation sensors.

In order to estimate the stability over time accurately, the RotSensor3C was placed in a quiet and stable environment to avoid external shakes and rotational noise, and thus the sensors only recorded pure earth rotation signals in this case. Then we calculated the recorded data of three orthogonal directions with ADEV method and the results are shown in Figure 2 in detail. According to the results, it is clear that performances of three components are pretty similar, and the angle random walk is approximately $2.67\times 10^{-4\circ }/\sqrt{h}$, which corresponds to a slope of −0.5 and takes its value at τ = 1 s in Figure 2a. The self-noise root power spectral density (PSD) is shown in Figure 2b, which indicates that the self noise is about $1.2\times 10^{-7}\;{\mathrm{rads}}^{-1}/\sqrt{\mathrm{Hz}}$ in the frequency ranging from 0.005 to 125 Hz. The root PSD curves are almost flat across the entire frequency band except high frequencies disturbed by environmental variation.

#### 2.2.2. Scale Factor Linearity

**Figure 3:**
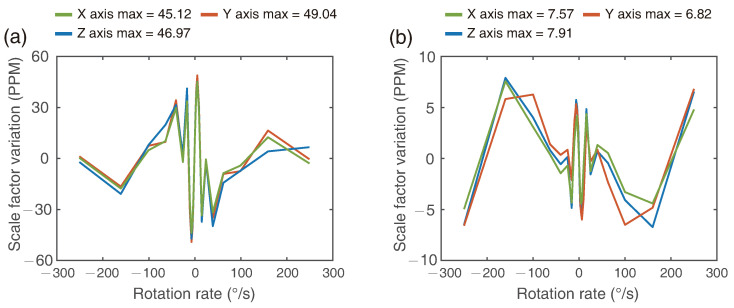
(**a**) Scale factor variation with input rotation rate before linearity compensation and (**b**) after linearity compensation.

An electrical system is designed and packed together with optical system to realize the periodic phase modulation and demodulation. The eigen frequency $f_{e}$ is related with the fiber coil length *L* and the refractive index *n* which can be expressed as $f_{e}=c/2nL$. In this 4km-long PM fiber coil, the modulation frequency $f_{m}=3f_{e}$ is about 73.5 kHz. Coherence demodulation method is adopted to obtain the first four harmonics amplitude $S_{1}$, $S_{2}$, $S_{3}$, $S_{4}$ at corresponding frequency points, which can be used to calculate the output rotation rate [21,22]. Typically, the scale factor is assumed to be frequency-independent, as the main noise in IFOGs is white noise. However, the frequency response of our electrical system is slightly different which will harms the scale factor linearity up to 50 ppm shown in Figure 3a, and thus the linearity compensation needs to be adopted.

We introduce four significant factors $\alpha_{i},\left(i=1,2,3,4\right)$ to compensate the frequency response, so the revised harmonics become $\alpha_{i}S_{i}$ and can be written as:

(2) $$\begin{array}{cc} \alpha_{1}S_{1} & =\left|2I_{0}J_{1}\left(\phi_{b}\right)\sin\left(\phi_{s}\right)\right|,\\ \alpha_{2}S_{2} & =\left|2I_{0}J_{2}\left(\phi_{b}\right)\cos\left(\phi_{s}\right)\right|,\\ \alpha_{3}S_{3} & =\left|2I_{0}J_{3}\left(\phi_{b}\right)\sin\left(\phi_{s}\right)\right|,\\ \alpha_{4}S_{4} & =\left|2I_{0}J_{4}\left(\phi_{b}\right)\cos\left(\phi_{s}\right)\right|, \end{array}$$

where the $I_{0}$ is the average light power; $J_{i}$ is the *i*th order Bessel functions of the first kind; $\phi_{b}$ and $\phi_{s}$ are the modulation depth and Sagnac phase shift, respectively. The $\phi_{b}$ can be obtained by

(3) $$\frac{\alpha_{1}S_{1}}{\alpha_{3}S_{3}}=\frac{J_{1}\left(\phi_{b}\right)}{J_{3}\left(\phi_{b}\right)}.$$

Then the rotation rate is given as:

(4) $$\Omega =\frac{\lambda c}{2\pi LD}\arctan\left|\frac{\alpha_{1}S_{1}}{\alpha_{2}S_{2}}\cdot \frac{J_{2}\left(\phi_{b}\right)}{J_{1}\left(\phi_{b}\right)}\right|.$$

In order to find the best factors to compensate the harmonics, the RotSensor3C was installed on a single-axis rotation table, and the table rotated clockwise and counter-clockwise at several certain rotation rates, respectively. For each rotation rate, the measurement continued 120 s to obtain an average output and the whole step would be repeated for these three orthogonal component. After collecting the harmonics, we used these data to calculate the factors: firstly set $\alpha_{1}=1$ to simplify the processing, and then the $\alpha_{2}$ and $\alpha_{3}$ would traverse from 0.9 to 1.1 to get temporary $\phi_{b}$ which can minimize the scale factor variation. The detail method of calculating the scale factor variation is in Appendix A. After getting the $\phi_{b}$, $\alpha_{2}$ and $\alpha_{3}$ and based on the presuppose that $\alpha_{1}=1$, the $\alpha_{4}$ could be obtained by

(5) $$\frac{\alpha_{2}S_{2}}{\alpha_{4}S_{4}}=\frac{J_{2}\left(\phi_{b}\right)}{J_{4}\left(\phi_{b}\right)}.$$

Then the factors would be written into the demodulation system to compensate the harmonics, and the RotSensor3C would rotate on the table again to determine whether the compensation took effect. The revised scale factor linearity is shown in Figure 3b. After the compensation, the scale factor variation with rotation rate ranging from $-250^{\circ }/s$ to $250^{\circ }/s$ is lower than 10 ppm.

#### 2.2.3. Sensor-Axis Orthogonality

In theory, the three sensitive axis of IFOGs should be ideally orthogonal to each other. However, due to the manufacturing limitations, the installation error is always existing, leading to cross coupling between the components of the sensor [13]. Therefore, we use a calibration method based on single-axis rate turntable to minimize the misalignment errors. The three scale factor of IFOGs are $k_{x},k_{y},k_{z}$, and the bias are $B_{x},B_{y},B_{z}$, respectively. The misalignment angle is expressed as $e_{ij}$, (i,j=x,y,z), which represents the angle error between sensors axis *i* and the body frame axis *j*. During the calibration process, the input standard rotation rates are $\omega_{x},\omega_{y},\omega_{z}$, and the output of IFOGs are $\Omega_{x},\Omega_{y},\Omega_{z}$. The error model of misalignment angles can be described as:

(6) $$\left(\begin{array}{c} \Omega_{x}\\ \Omega_{y}\\ \Omega_{z} \end{array}\right)=\left(\begin{array}{ccc} k_{x} & k_{x}e_{xy} & k_{x}e_{xz}\\ k_{y}e_{yx} & k_{y} & k_{y}e_{yz}\\ k_{z}e_{zx} & k_{z}e_{zy} & k_{z} \end{array}\right)\cdot \left(\begin{array}{c} \omega_{x}\\ \omega_{y}\\ \omega_{z} \end{array}\right)+\left(\begin{array}{c} B_{x}\\ B_{y}\\ B_{z} \end{array}\right),$$

by utilizing the method in Reference [23], the 3 × 3 misalignment matrix M is derived as:

(7) $$M=\left(\begin{array}{ccc} 0.991775185 & 0.000297482 & -0.000462613\\ 0.001192965 & 0.99400358 & -0.00073276\\ -0.000246073 & -0.000558209 & 0.992409135 \end{array}\right).$$

**Figure 4:**
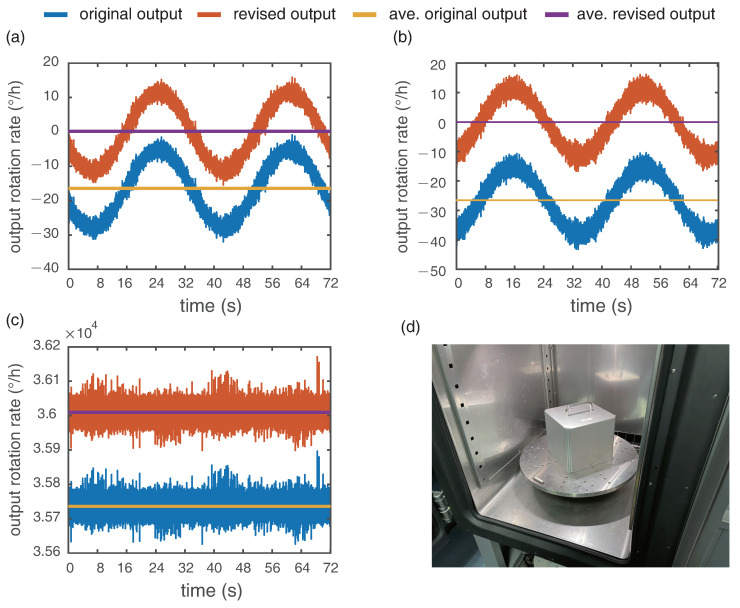
The output of three components with and without misalignment matrix: (**a**) x axis; (**b**) y axis and (**c**) z axis; (**d**) single-axis rotation table inside the temperature chamber.

The results with misalignment matrix to compensate the cross-coupling errors are shown in Figure 4. The latitude of our laboratory is 39.991844 so the local corresponding vertical rotation rate of earth is $9.667^{\circ }/h$. As the RotSensor3C is placed with *z* axis vertically and the table rotation rate is $10^{\circ }/s$, the output of *z* axis should be 36,009.6${}^{\circ }/h$ (added the local earth rotation rate). The outputs $\Omega_{i},\left(i=x,y\right)$ are related to the respective angles $\theta_{i}$ between the sensor axis and north–south (NS) direction, which can be expressed as:

(8) $$\Omega_{i}=\Omega_{h}\times \sin\theta_{i}$$

where $\Omega_{h}$ is the local horizontal rotation rate. Therefore, under the situation of $10^{\circ }/s$ vertical rotation, the outputs of *x* and *y* axis should be trigonometric curves with a peak value of our local horizontal rotation rate $11.52^{\circ }/h$ and an average value of zero with a cycle 36 s in theory. The revised output in Figure 4 correspond to the theory, which indicates the records of RotSensor3C are quantified.

As a new designed 3C rotational seismometer, here we select another two kinds of well-known three components rotation sensors (R-2, BlueSeis3A) [24] and draw a comparison shown as the Table 1. Notice that the Rotsensor3C and R-2 have no battery inside, so the comparison of dimensions and weight just for reference only.

## 3. Near Field Explosion Seismic Test

With the above detail laboratory tests, the characteristics of RotSensor3C are fully demonstrated and some practical applications have been carried out. Explosion seismic is a common method to generate seismic waves and determine the sensors performance. Therefore we organized a near field explosive experiment together with translational sensors to test our RotSensor3C and verify the related theory meanwhile.

**Figure 5:**
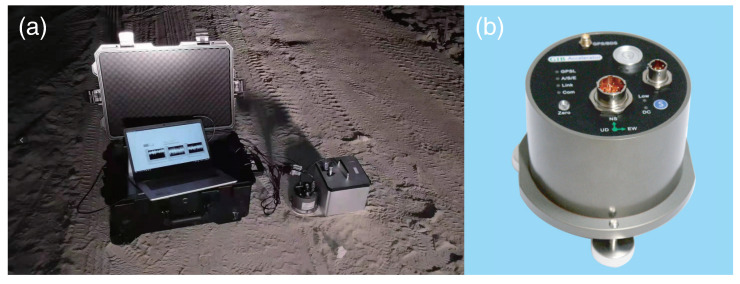
Experimental setup for a temporary single station six-component measurements: (**a**) two instruments installation scheme; (**b**) translational seismometer G1B.

**Figure 6:**
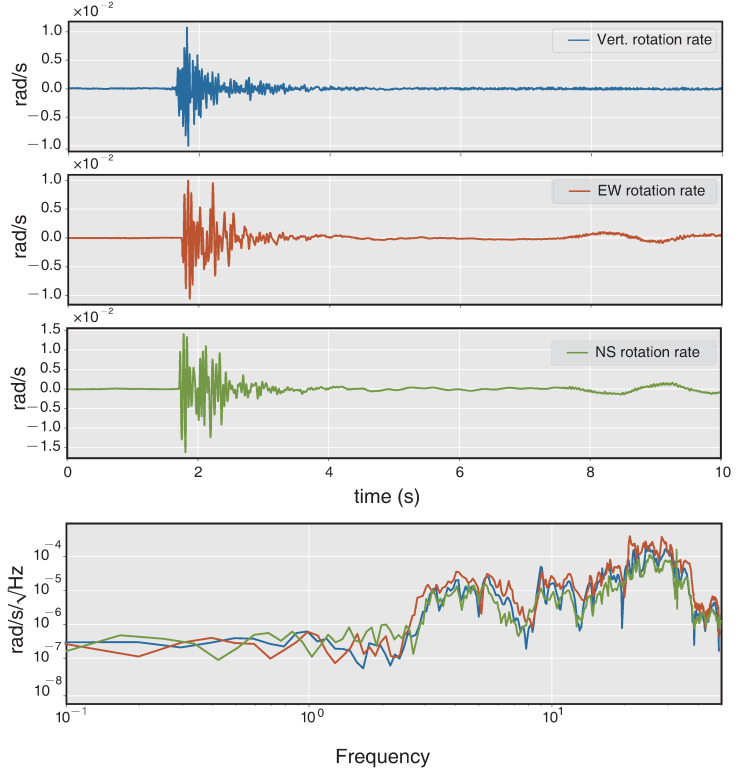
Amplitudes and spectra of rotation rate recorded by the RotSensor3C.

**Figure 7:**
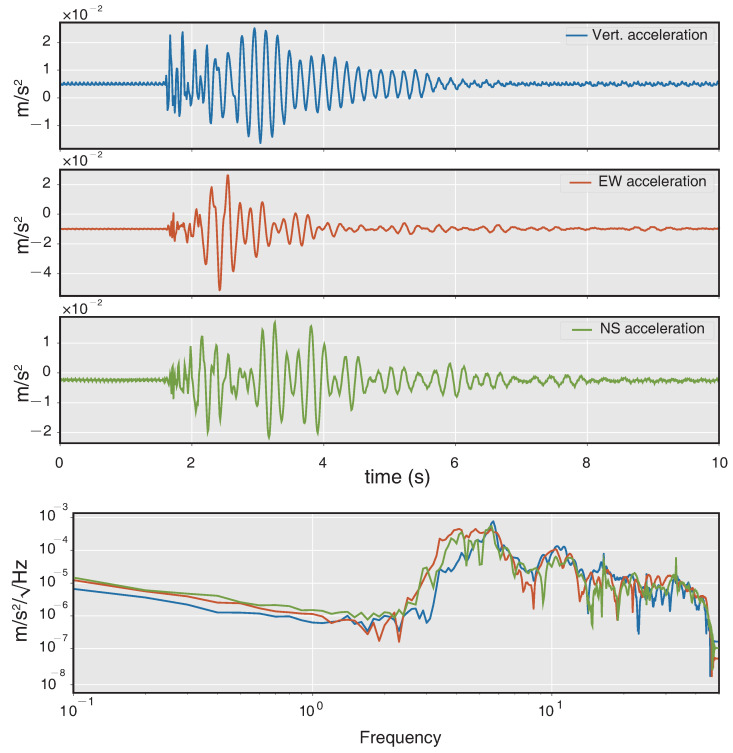
Amplitudes and spectra of translational acceleration recorded by the G1B.

The experimental setup is shown in Figure 5a, all the sensors were co-located on a single rigid base. The distance between the two instruments is about 3 cm, and therefore the comprehensive system can be considered as a single-station measurement to record signals that originate from same ground motions. As this test is aimed at analyzing near field waves, the distance between the explosion point and measure station is set to ∼150 m. The model of the three-component translational seismometer shown in Figure 5b is G1B developed by China Earthquake Administration. Figure 6 shows the amplitudes and spectra of rotational velocity recorded by the RotSensor3C, the peak rotation velocity is 0.016 rad/s for the NS component (Rn), and the amplitudes of the other two components (Rv/Re) are slightly lower. The spectra of them are in great consistency which shows that the dominant frequency band of rotational seismic waves is from about 2 to 40 Hz. The amplitudes and spectra of translational acceleration recorded by the G1B are shown in Figure 7, the peak ground acceleration is 0.056 $m/s^{2}$ for the EW component (Ae) and the spectra are also pretty similar to each other indicating that the dominant frequency band of ground acceleration is from about 2 to 20 Hz, which differ from the rotational components at high frequency.

**Figure 8:**
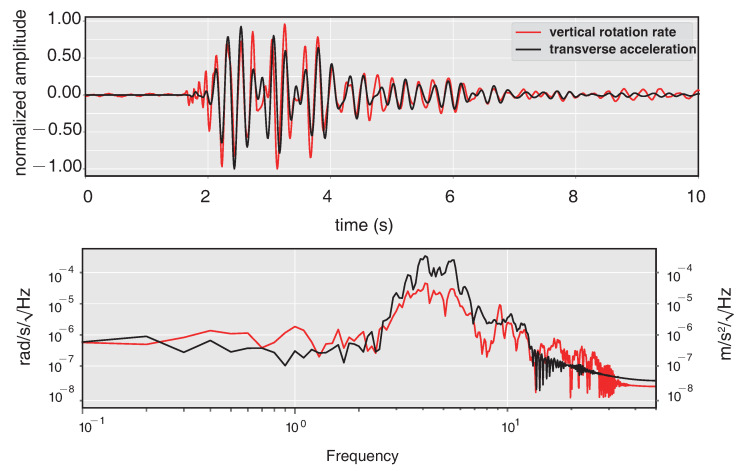
Normalized amplitude and spectra of vrtical rotation rate and transverse acceleration after band-pass filtered.

**Figure 9:**
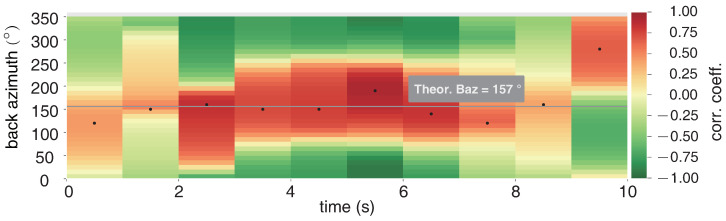
Back-azimuths (BAzs) estimation with CC method.

**Figure 10:**
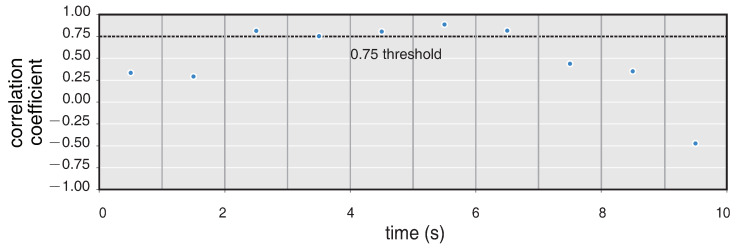
Cross-correlation coefficients between the transverse acceleration and rotation rate computed for a sliding time window.

The six-component measurement provides a new method to estimate the additional information of seismic waves, such as back-azimuths (BAz) and phase velocity. Here we use the rotational seismology tools of a community platform (www.seismo-live.org, accessed on 1 March 2021) to process the vertical rotation (Rv) and two acceleration components EW (Ae) and NS (An). Firstly, all the components are band-pass filtered between 0.1 and 10 Hz, as this frequency band of the near-field earthquake is what we concerned. Then the two translation components Ae and An are rotated into the transverse component (At) based on a trial BAz and calculate the cross-correlation of At and Rv. Since At and Rv are both result from the same ground motion, they will reach a maximum cross-correlation when the trial BAz coincides with the true one [25]. The normalized amplitude and spectra of transverse acceleration rotated by an appropriate BAz and vertical rotation are shown in Figure 8, in which the normalized amplitude over time show a strong correlation of them. Moreover, according to the PSD analysis in Figure 8, both background noise and the signal spectrum appear to be highly correlated with each other. A time step of 1s is applied to continuously estimate BAzs and CC along the whole 10s seismogram. Figure 9 shows the whole BAzs indicated by the black dots of each time window, which is consistent with the theoretical BAz (∼157${}^{\circ }$) shown with the gray line. Figure 10 shows the correlation coefficients of each sliding time window, as the seismic signals mainly distribute in 2∼7 s, the correlation coefficients > 0.75 focus on the same time parts.

**Figure 11:**
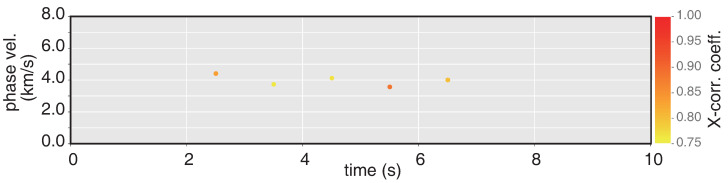
Phase velocity of SH-type waves for 1s time step with correlation coefficients > 0.75.

Besides the BAzs, according to the relationships between transverse acceleration and vertical rotation [26,27], phase-velocity can also be estimated by the following equation:

(9) $$V_{SH}=\left|\frac{\mathrm{At}}{2\times \mathrm{Rv}}\right|$$

(10) $$V_{SV}=\left|\frac{\mathrm{Av}}{\mathrm{Rt}}\right|$$

in which the $V_{SH}$ is the apparent phase velocity of horizontal polarized SH-type, the $V_{SV}$ is the apparent phase velocity of vertical polarized SV-type. The detail explanation of the above relationship is in Appendix B. Figure 11 shows the phase velocity of SH-type waves derived from amplitude ratios of acceleration and rotation rate with correlation coefficients > 0.75. These results are in great agreement with the related theory, and proves the quality of the RotSensor3C which can offer a unique method to obtain significant information.

## 4. Conclusions and Prospects

In this paper, a three components rotational seismometer RotSensor3C based on open loop IFOGs is designed and demonstrated. According to the laboratory results, the RotSensor3C has a broadband frequency response from 0.005 Hz to 125 Hz, and its self-noise is about $1.2\times 10^{-7}\;{\mathrm{rads}}^{-1}/\sqrt{\mathrm{Hz}}$. Moreover, because of the linearity compensation, the scale factor linearity is improved from 50 ppm to lower than 10 ppm at certain rotation rate, and the misalignment matrix makes the output rotation rate more accurate. Explosion seismic test confirmed the practicability of this new rotation sensors. Our test results prove that the RotSensor3C is quite capable of recording rotational seismic data. Meanwhile, more improvements have been planned to the next generation.

As our first 3C rotational seismometer, we choice the single polarized configuration. The sensitivity of RotSensor3C will be improved next by utilize the sensitivity-enhanced dual-polarized configuration, and in theory the self-noise would be reduced by 8 times at the same size [28]. Moreover, as the dual-polarized configuration is insensitive to temperature variations and magnetic field due to the two orthogonal polarizations compensation effect [29,30], the stability and accuracy will be improved meanwhile. The translation components are significant for completely recording seismic waves as well, so the relevant research is moving on and the next six-component seismometer is on our plan.

## Figures and Tables

**Table 1:** Comparison of main characteristics of three fiber optic rotational seismometer.

Performance	RotSensor3C	R-2	BlueSeis3A
Sensor self-noise $\left({\mathrm{rads}}^{-1}/\sqrt{\mathrm{Hz}}\right)$	$1.2\times 10^{-7}$	$6.0\times 10^{-8}$	$2.0\times 10^{-8}$
Frequency range	0.005–125 Hz	0.03–50 Hz	0.001–100 Hz
Dynamic range	152 dB	117 dB	135 dB
Scale factor linearity	<10 ppm	No data	<20 ppm
Dimensions (L × W × H)	190 × 190 × 165 mm	120 × 120 × 102 mm	300 × 300 × 280 mm
	(No battery)	(No battery)	
Weight	4.5 kg (No bat.)	9.53 kg (No bat.)	20 kg

## Data Availability

Not applicable.

## Appendix A

The ${\bar{F}}_{j}$ is the average gyroscope output caused by the input rotate rate $\Omega_{j}$ of single-axis table and the earth rotation ${\bar{F}}_{r}$. The linear relationship between them can be expressed as

(A1) $$F_{j}={\bar{F}}_{j}-{\bar{F}}_{r}=K\cdot \Omega_{j}+F_{0},$$

where $F_{j}$ presents the output without earth rotation which totally originates from input table rotation, and *K* is the scale factor, $F_{0}$ is the inherent offset of gyroscope which is typically smaller than $10^{-6\circ }/h$. They can be obtained by

(A2) $$K=\frac{\sum_{j=1}^{M}\Omega_{j}\cdot F_{j}-\frac{1}{M}\sum_{j=1}^{M}\Omega_{j}\cdot \sum_{j=1}^{M}F_{j}}{\sum_{j=1}^{M}\Omega_{j}^{2}-\frac{1}{M}{\left(\sum_{j=1}^{M}\Omega_{j}\right)}^{2}},$$

(A3) $$F_{0}=\frac{1}{M}\sum_{j=1}^{M}F_{j}-\frac{K}{M}\sum_{j=1}^{M}\Omega_{j},$$

where *M* is the number of rotation rate. Then a fitting output ${\hat{F}}_{j}$ of the corresponding gyroscope is calculated and written as:

(A4) $${\hat{F}}_{j}=K\cdot \Omega_{j}+F_{0}.$$

Therefore the output errors of each rotation rate point between the original output and fitting output can be obtained by

(A5) $$\delta_{j}=\frac{{\hat{F}}_{j}-F_{j}}{|F_{m}|},$$

where $F_{m}$ is the range of test rotation rate, and according to the errors of every rotation rate, the scale factor linearity $K_{n}$ is defined as:

(A6) $$K_{n}=\max\left|\delta_{j}\right|.$$

## Appendix B

In the framework of classical elasticity, with the assumption of infinitesimal deformations, a function of space u=u(x,t) is used to denote the vector distance of a particle at the position x. For a point x and the point x+δx in its vicinity, after a distortion δx, the new locations of two points are (x+u(x)) and x+δx+u(x+δx), respectively. Therefore, the relative location of two points can be written as [31]:

(A7) δx+δu=x+δx+u(x+δx)−(x+u(x)).

Since |δx| is arbitrarily small, we can expand u(x+δx) as u+(δx·∇)u by neglecting high order terms. It follows that δu is related to both the gradients of u and the original line element δx via [31]

(A8) δu=(δx·∇)u,

and we can use the gradient tensor to describe the displacement δu as:

(A9) $$\left(\begin{array}{c} \delta u_{x}\\ \delta u_{y}\\ \delta u_{x} \end{array}\right)=\left(\begin{array}{ccc} \frac{\partial u_{x}}{\partial x} & \frac{\partial u_{x}}{\partial y} & \frac{\partial u_{x}}{\partial z}\\ \frac{\partial u_{y}}{\partial x} & \frac{\partial u_{y}}{\partial y} & \frac{\partial u_{y}}{\partial z}\\ \frac{\partial u_{z}}{\partial x} & \frac{\partial u_{z}}{\partial y} & \frac{\partial u_{z}}{\partial z} \end{array}\right)\left(\begin{array}{c} \delta x\\ \delta y\\ \delta z \end{array}\right).$$

The Equation (A9) can be rewritten as:

(A10) $$\begin{array}{cc} \left(\begin{array}{c} \delta u_{x}\\ \delta u_{y}\\ \delta u_{x} \end{array}\right)\; & =\left(\begin{array}{ccc} \frac{\partial u_{x}}{\partial x} & 0 & 0\\ 0 & \frac{\partial u_{y}}{\partial y} & 0\\ 0 & 0 & \frac{\partial u_{z}}{\partial z} \end{array}\right)\left(\begin{array}{c} \delta x\\ \delta y\\ \delta z \end{array}\right)\\ \; & +\left(\begin{array}{ccc} 0 & \frac{1}{2}\left(\frac{\partial u_{x}}{\partial y}+\frac{\partial u_{y}}{\partial x}\right) & \frac{1}{2}\left(\frac{\partial u_{x}}{\partial z}+\frac{\partial u_{z}}{\partial x}\right)\\ \frac{1}{2}\left(\frac{\partial u_{y}}{\partial x}+\frac{\partial u_{x}}{\partial y}\right) & 0 & \frac{1}{2}\left(\frac{\partial u_{y}}{\partial z}+\frac{\partial u_{z}}{\partial y}\right)\\ \frac{1}{2}\left(\frac{\partial u_{z}}{\partial x}+\frac{\partial u_{x}}{\partial z}\right) & \frac{1}{2}\left(\frac{\partial u_{z}}{\partial y}+\frac{\partial u_{y}}{\partial z}\right) & 0 \end{array}\right)\left(\begin{array}{c} \delta x\\ \delta y\\ \delta z \end{array}\right)\\ \; & +\left(\begin{array}{ccc} 0 & \frac{1}{2}\left(\frac{\partial u_{x}}{\partial y}-\frac{\partial u_{y}}{\partial x}\right) & \frac{1}{2}\left(\frac{\partial u_{x}}{\partial z}-\frac{\partial u_{z}}{\partial x}\right)\\ \frac{1}{2}\left(\frac{\partial u_{y}}{\partial x}-\frac{\partial u_{x}}{\partial y}\right) & 0 & \frac{1}{2}\left(\frac{\partial u_{y}}{\partial z}-\frac{\partial u_{z}}{\partial y}\right)\\ \frac{1}{2}\left(\frac{\partial u_{z}}{\partial x}-\frac{\partial u_{x}}{\partial z}\right) & \frac{1}{2}\left(\frac{\partial u_{z}}{\partial y}-\frac{\partial u_{y}}{\partial z}\right) & 0 \end{array}\right)\left(\begin{array}{c} \delta x\\ \delta y\\ \delta z \end{array}\right) \end{array}.$$

The first two parts of Equation (A10) are related with the infinitesimal strain tensor ε. The third term corresponding to the infinitesimal rotation tensor which can also be expressed as rotation vector ω [7], and thus we can simplify the Equation (A10) to

(A11) δu=εδx+ω×δx,

where the rotation vector ω is

(A12) $$\omega =\frac{1}{2}\nabla \times u\left(x\right).$$

Now we consider a transversely polarized SH-type (e.g., SH and Love), with displacement $u\left(x,y,z,t\right)=\left(0,u_{y}\left(t-x/V_{SH}\right),0\right)$, where $V_{SH}$ is the apparent horizontal phase velocity. The rotational vector ω can be obtained according the Equation (A12) and written as [32]:

(A13) $$\begin{array}{cc} \omega & =\frac{1}{2}\nabla \times u\left(x\right)\\ \; & =(0,0,{\dot{u}}_{y}\left(t-x/V_{SH}\right)/2V_{SH}) \end{array},$$

in which the corresponding z-component of rotation rate is $\omega_{z}={\ddot{u}}_{y}\left(t-x/V_{SH}\right)/2V_{SH}$. This implies that under the assumptions that at any time rotation rate and transverse acceleration are in phase, the phase velocity can be calculated by [32]

(A14) $$\left|\frac{{\ddot{u}}_{y}\left(t-x/V_{SH}\right)}{2\times \omega_{z}}\right|=V_{SH}.$$

Besides the SH-type waves, the phase velocity of SV-type (e.g., SV and Rayleigh) can also be estimated with the similar method [27]:

(A15) $$\left|\frac{{\ddot{u}}_{z}}{\omega_{y}}\right|=V_{SV},$$

where ${\ddot{u}}_{z}$ is the acceleration of the particle motions along the Z-axis, $\omega_{y}$ is the rotation rate of Y-axis, and $V_{SH}$ is the apparent phase velocity of Rayleigh wave.

## References

[1] Cochard A., Igel H., Schuberth B., Suryanto W., Velikoseltsev A., Schreiber U., Wassermann J., Scherbaum F., Vollmer D. (2006). Rotational motions in seismology: Theory, observation, simulation. Earthquake Source Asymmetry, Structural Media and Rotation Effects.

[2] Lee W.H., Igel H., Trifunac M.D. (2009). Recent advances in rotational seismology. Seismol. Res. Lett..

[3] Pham N.D. (2009). Rotational Motions in Seismology: Theory, Observation, Modeling. Ph.D. Thesis.

[4] Guéguen P., Guattari F., Aubert C., Laudat T. (2021). Comparing Direct Observation of Torsion with Array-Derived Rotation in Civil Engineering Structures. Sensors.

[5] Bońkowski P.A., Zembaty Z., Minch M.Y. (2019). Engineering analysis of strong ground rocking and its effect on tall structures. Soil Dyn. Earthq. Eng..

[6] Guéguen P., Astorga A. (2021). The torsional response of civil engineering structures during earthquake from an observational point of view. Sensors.

[7] Sollberger D., Igel H., Schmelzbach C., Edme P., van Manen D.J., Bernauer F., Yuan S., Wassermann J., Schreiber U., Robertsson J.O. (2020). Seismological Processing of Six Degree-of-Freedom Ground-Motion Data. Sensors.

[8] Igel H., Cochard A., Wassermann J., Flaws A., Schreiber U., Velikoseltsev A., Pham Dinh N. (2007). Broad-band observations of earthquake-induced rotational ground motions. Geophys. J. Int..

[9] Huang B., Liu C., Lin C., Wu C., Lee W. Measuring mid-and near-field rotational ground motions in Taiwan. Presented at the 2006 Fall AGU Meeting.

[10] Liu C.C., Huang B.S., Lee W.H., Lin C.J. (2009). Observing rotational and translational ground motions at the HGSD station in Taiwan from 2007 to 2008. Bull. Seismol. Soc. Am..

[11] Kurzych A., Jaroszewicz L.R., Krajewski Z., Teisseyre K.P., Kowalski J.K. (2014). Fibre optic system for monitoring rotational seismic phenomena. Sensors.

[12] Velikoseltsev A., Schreiber K.U., Yankovsky A., Wells J.P.R., Boronachin A., Tkachenko A. (2012). On the application of fiber optic gyroscopes for detection of seismic rotations. J. Seismol..

[13] Bernauer F., Wassermann J., Guattari F., Frenois A., Bigueur A., Gaillot A., de Toldi E., Ponceau D., Schreiber U., Igel H. (2018). BlueSeis3A: Full characterization of a 3C broadband rotational seismometer. Seismol. Res. Lett..

[14] Jaroszewicz L.R., Kurzych A., Krajewski Z., Marć P., Kowalski J.K., Bobra P., Zembaty Z., Sakowicz B., Jankowski R. (2016). Review of the usefulness of various rotational seismometers with laboratory results of fibre-optic ones tested for engineering applications. Sensors.

[15] Post E.J. (1967). Sagnac effect. Rev. Mod. Phys..

[16] Vali V., Shorthill R. (1976). Fiber ring interferometer. Appl. Opt..

[17] Li Y., Cao Y., He D., Wu Y., Chen F., Peng C., Li Z. (2019). Thermal phase noise in giant interferometric fiber optic gyroscopes. Opt. Express.

[18] Allan D.W. (1966). Statistics of atomic frequency standards. Proc. IEEE.

[19] El-Sheimy N., Hou H., Niu X. (2007). Analysis and modeling of inertial sensors using Allan variance. IEEE Trans. Instrum. Meas..

[20] Lefevre H.C. (2014). The Fiber-Optic Gyroscope.

[21] Böhm K., Marten P., Weidel E., Petermann K. (1983). Direct rotation-rate detection with a fibre-optic gyro by using digital data processing. Electron. Lett..

[22] Gronau Y., Tur M. (1995). Digital signal processing for an open-loop fiber-optic gyroscope. Appl. Opt..

[23] Zhao X.Q., Chao D.H., Song L.L. (2014). Calibration method for angle speed sensor based on single-axis rate turntable. Transducer Microsyst. Technol..

[24] Kislov K., Gravirov V. (2021). Rotational Seismology: Review of Achievements and Outlooks. Seism. Instrum..

[25] Yuan S., Simonelli A., Lin C.J., Bernauer F., Donner S., Braun T., Wassermann J., Igel H. (2020). Six Degree-of-Freedom Broadband Ground-Motion Observations with Portable Sensors: Validation, Local Earthquakes, and Signal Processing. Bull. Seismol. Soc. Am..

[26] Suryanto W., Igel H., Wassermann J., Cochard A., Schuberth B., Vollmer D., Scherbaum F., Schreiber U., Velikoseltsev A. (2006). First comparison of array-derived rotational ground motions with direct ring laser measurements. Bull. Seismol. Soc. Am..

[27] Lin C.J., Huang H.P., Pham N.D., Liu C.C., Chi W.C., Lee W.H. (2011). Rotational motions for teleseismic surface waves. Geophys. Res. Lett..

[28] He D., Cao Y., Zhou T., Peng C., Li Z. (2020). Sensitivity enhancement through RIN suppression in dual-polarization fiber optic gyroscopes for rotational seismology. Opt. Express.

[29] Luo R., Li Y., Deng S., He D., Peng C., Li Z. (2017). Compensation of thermal strain induced polarization nonreciprocity in dual-polarization fiber optic gyroscope. Opt. Express.

[30] Liu P., Li X., Guang X., Xu Z., Ling W., Yang H. (2017). Drift suppression in a dual-polarization fiber optic gyroscope caused by the Faraday effect. Opt. Commun..

[31] Aki K., Richards P.G. (2002). Quantitative Seismology.

[32] Igel H., Schreiber U., Flaws A., Schuberth B., Velikoseltsev A., Cochard A. (2005). Rotational motions induced by the M8. 1 Tokachi-oki earthquake, September 25, 2003. Geophys. Res. Lett..

